# Deep vein thrombosis in shoulder arthroplasty – a prospective study

**DOI:** 10.1186/1471-2474-14-139

**Published:** 2013-04-18

**Authors:** Thayur R Madhusudhan, Amit Sinha, David Widdowson

**Affiliations:** 1 Department of Orthopaedics and trauma, Betsi Cadwaldr University Health Board [BCUHB], central division, Rhyl, North Wales LL185UJ, UK; 2 Shoulder and Elbow services, Department of Trauma and Orthopaedics, Betsi Cadwaldr University Health Board [BCUHB], central division, Rhyl, North Wales LL185UJ, UK; 3 Department of Radiology, Betsi Cadwaldr University Health Board [BCUHB], central division, Rhyl, North Wales LL185UJ, UK

**Keywords:** Deep vein thrombosis, Shoulder arthroplasty, Doppler scans

## Abstract

**Background:**

Shoulder arthritis of varied aetiology is often disabling and patients seek treatment for pain relief and restricted movements. Though non operative measures in the form of analgesics, physiotherapy and joint injections offer satisfactory results in the early stages; operative treatment in the form of joint replacements becomes necessary in late and advanced stages. The above operations are being performed more frequently in the recent years across the National Health Service [NHS] in the UK with increasing success in specialised units and the outcome of the operation is often rewarding. In addition to the other complications, risks of Deep vein thrombosis [DVT] and pulmonary embolism [PE] exists. Available evidence suggests a low incidence but the true risk has only been partially addressed. The final decision to consider thromboprophylaxis rests with the operating surgeon. It is important to carefully balance the clinical decision of thromboprophylaxis and bleeding with wound complications, which add considerable morbidity and mortality. To define the risk of DVT in this subgroup of patients is the initial step to enable better use of resources and achieve cost effectiveness. This we believe will provide robust evidence to help formulate guidelines for thromboprophylaxis in shoulder arthroplasty.

**Methods/design:**

The aim will be to determine whether shoulder arthroplasties carry a risk of DVT. A cohort of 100 consecutive patients being considered for shoulder arthroplasty for degenerative arthritis, rotator cuff arthropathy, inflammatory arthropathy including rheumatoid arthritis will be prospectively included for the study. All eligible patients will be assessed clinically and screened for DVT in all 4 limbs both pre and postoperative with Doppler scans within a 6 week perioperative period. We aim to include the reasons for non inclusion of eligible patients and patient’s perspective of their general well being in relation to DVT.

**Discussion:**

We present the risk of DVT associated with shoulder arthroplasties to establish a good quality evidence for thromboprophylaxis. The study is underway and we would further be able to define whether the general risk factors for DVT are relevant to shoulder replacements.

## Study aim

We aim to improve the quality of available evidence of whether shoulder arthroplasties carry a risk of DVT. This will help us to determine the need for thromboprophylaxis.

## Background

Shoulder arthritis of varied aetiology is often disabling and patients seek treatment for pain relief and restricted movements. Though non operative measures in the form of analgesics, physiotherapy and joint injections offer satisfactory results in the early stages; operative treatment in the form of joint replacements becomes necessary in late and advanced stages. The above operations are being performed more frequently in the recent years across the National Health Service [NHS] in the UK with increasing success in specialised units and the outcome of the operation is often rewarding. In addition to the other complications, risks of Deep vein thrombosis [DVT] and pulmonary embolism [PE] exists. Several case reports, retrospective studies, surgeon surveys, database reviews and results from the National joint registry, UK reports a low incidence [1-6] of DVT. The true risk of DVT has only been partially addressed in one study [7]. NICE guidelines [8] recommend risk assessment and offering mechanical and or chemoprophylaxis routinely for all patients undergoing upper limb surgery. We intend improving the quality of available evidence. The final decision to consider thromboprophylaxis rests with the operating surgeon. It is important to carefully balance the clinical decision of thromboprophylaxis and bleeding with wound complications, which add considerable morbidity and mortality. To define the risk of DVT in this subgroup of patients is the initial step to enable better use of resources and achieve cost effectiveness. This we believe will provide robust evidence to help formulate guidelines for thromboprophylaxis in shoulder arthroplasty.

## Methods

We intend to conduct a prospective cohort study to assess the risk of DVT in shoulder arthroplasties. This includes systematic collection of data and study of baseline characteristics from all eligible patients, irrespective of their participation in the study. As detailed below, the study emphasizes standard protocols within the NHS trust with well defined patient management pathways. This has been possible following consultations and interactions with the medical and haematology departments within the NHS trust.

The patient continues to be under the care of the orthopaedic team until safe discharge and therefore we anticipate we will be able to complete the study with a less likelihood of patients ‘dropping out’ of the study. All untoward events including mortality and the relevant reasons will be documented. The study group is representative of the population which will therefore not threaten the external validity of the study.

### Inclusion criteria

All patients with suitable clinical symptoms and signs of arthritis who are anaesthetic fit for shoulder arthroplasty will be included in the study. Shoulder arthroplasty includes total shoulder replacement, hemiarthroplasty and reverse shoulder arthroplasty. The main indications for shoulder arthroplasty will be degenerative arthritis and rotator cuff arthropathy.

### Exclusion criteria

• Patients positive for DVT on pre operative Doppler scanning

• Patients with co morbidities requiring anticoagulation, including previous malignancies and previously identified coagulation disorders.

• Patients who will not be able to follow up for other reasons.

### Brief details of the practical arrangements for recruitment

All patients will be clinically examined and assessment is completed with standard views of plain radiographs preoperatively. The proposed procedure including the nature of the operation contemplated, risks, routine postoperative care including physiotherapy and follow-up, expected outcome will be discussed. The purpose of the proposed study will be discussed and if the patient is willing, shall be included in the study. An information leaflet which will briefly describe the nature of the problem investigated will be given to the patients included for the study. The patients are then consented for inclusion in the study in addition to the site specific consent for the operative procedure. With the patient’s permission, letters will be sent to their General practitioners [GP] to inform them of participation. We will request patient permission to allow us to ask their GP to provide information on their contact details if we are unable to contact them directly.

### Pre operative work up

Blood tests including coagulation profile [Fibrinogen levels, prothrombin time, and Activated prothrombin time], metabolic work up for liver and renal functions, and additional investigations depending on the co-morbidities will be completed as part of the pre operative assessment. All patients will be screened with pre operative 4 limbs Doppler scans within 3 weeks from the date of the operation. Patients with asymptomatic DVT detected on Doppler scanning, will be excluded from the study and further be referred to the concerned medical team for further management. Those patients who are negative for DVT on screening will proceed for the operative procedure which will be as per prescribed standards. With screening, we have therefore obliviated the need for a separate control group.

### Peri-operative management

Peri-operative management will include anaesthesia, analgesia, antibiotic administration and shoulder arthroplasty performed in beach chair position as per routine standards. No patient will be prescribed chemical thromboprophylaxis. However all routine mechanical intra-operative precautions will be followed.

### Post operative management

All patients will be assessed and physiotherapy will be supervised by a qualified physiotherapist before discharge. We promote the need to encourage patients to perform home exercises and the physiotherapist will provide all the guidance and illustrative material. The operating surgeon or his deputy will request postoperative 4 limb Doppler scans before discharge along with arrangements for follow up. We aim to arrange Doppler scans within 4 weeks postoperative period, in anticipation that most patients would be clinically comfortable to be able to co operate and also considering the existing load within the NHS framework. Similar prospective study reports a lower incidence of DVT at 6 weeks in contrast to a higher incidence at 2 days on scans. This may reflect a natural trend for clots to dissolve within the body system [7]. We therefore have chosen to arrange postoperative scans around 3–4 weeks to provide meaningful information about the occurrence of DVT.

All radiology scans will be performed by suitably qualified and trained radiographers. All reports will be validated by a consultant radiologist and will be uploaded and stored on the hospital radiology software system which will be used for data analysis. All patient data will be stored confidential as per the NHS recommendations. Doppler scans positive for DVT in the postoperative period will be managed as per agreed and established guidelines of DVT. All discharged patients will be followed up for any untoward incidents before the scheduled follow up, with a communication sent to the general practitioner. At the scheduled postoperative follow up, the scan results will be reviewed and discussed with the patient.

### Ethics and impact on participants

MREC approval has been obtained from North West Wales research ethics committee [Ref: 08/WNo01/35]. As outlined above we are looking to provide good quality evidence and the investigation methods employed have been well established as safe and effective for screening purposes. We therefore do not anticipate any additional risk to the patients or compromising on the safety, with all standardised protocols followed throughout the period of the study.

The participant informant sheet [PIS] has been developed to provide a balanced account of the problem which is being addressed and clearly explain the objectives of the study without compromising on the quality of care. This will be the case whether the participant decides to enter the study or decides not to consent. Written informed consent is obtained from all recruited subjects. All relevant documentation will be stored along with the patient notes as stipulated within the NHS framework.

### Data collection

Basic scientific data for all patients recruited will be done systematically and documented. Also we would note, wherever possible, the reasons for patient opting out of the study either at entry or midway. The relevant data collected will include ethnicity, education status, hand dominance, co morbidities, smoking, patient contact details, GP name and surgery. The process will be made simplistic and easily retrievable. The data collection for the above problem stops at the scheduled first visit postoperative when we should have the scan results available.

### Sample size

The primary question addressed in this study is whether shoulder arthroplasty increases the risk of deep vein thrombosis. From previous available data, it is expected that the rate of DVT will be approximately 13% in shoulder arthroplasty [7]. With this assumed value and with a CI of 8–21, we propose to include 100 participants for shoulder arthroplasty to achieve a power of over 80% [83% actual] of finding a significant effect at 5% level. The actual numbers will be probably less than the projected and therefore our estimate of 10% drop-outs is purposely pessimistic for sample size calculations.

### Period of study and data dissemination

The study is underway and our recruitment period is 24 months. We anticipate that recruitment will be challenging particularly across the other sites. We shall endeavour to achieve the target within the stipulated period. To achieve our required numbers, we may include other similar NHS sites in the study depending on the progress and this will be in accordance with the stipulated regulations. Study completion includes submission of the final report to the BCUHB research committee, the funders to the project and dissemination of the findings through relevant national and international conferences and peer reviewed publications.

## Discussion

This article is the version 3 [24/11/2009] of the protocol for the study. This has been approved by the ethics committee after various suggestions and adjustments were made to the original protocol. Many changes were made in keeping with the required standards of research and clarifications in wording the PIS and the consent form. All changes have resulted in increased clarity and we hope this will increase the effectiveness of the study.

## Competing interests

The authors declare that they have no competing interests.

## Authors’ contributions

All authors have contributed to the design and development of the study protocol. AS is the chief investigator and has the overall responsibility of the study. TRM and DW are co investigators. TRM designed the study and was involved in writing up the study protocol, PIS, consent, developing standardised protocols of patient management pathways, organising the necessary investigations, data collection and interpretation, statistical analysis, and dissemination including writing the manuscript. DW organised and reported all the Doppler scans. All authors have read and approved the manuscript.

## Pre-publication history

The pre-publication history for this paper can be accessed here:

http://www.biomedcentral.com/1471-2474/14/139/prepub
